# Load Transfer along Continuous Collagen Fibers Reduces the Importance of Wall Thickness Variations

**Published:** 2025-09-29

**Authors:** Yamnesh Agrawal, Masoud Zamani, James R. Thunes, Spandan Maiti, Anne M. Robertson

**Affiliations:** (1) Mechanical Engineering & Materials Science, University of Pittsburgh, Pittsburgh, PA, USA; (2) ANSYS Canada Ltd, Waterloo, ON, Canada; (3) Bioengineering, University of Pittsburgh, Pittsburgh, PA, USA; (4) Chemical and Petroleum Engineering, University of Pittsburgh, Pittsburgh, PA, USA

## Abstract

The mechanical response of biological soft tissues is influenced by wall heterogeneity, including spatial variations in wall thickness. Traditional models for homogeneous soft tissues under uniaxial loading predict higher stretch and stress in thinner regions. In fact, large gradients in stretch and stress are predicted to be induced by spatial variations in wall thickness. In prior studies, the role of collagen fibers in regions of thickness transition has been largely neglected or only considered in terms of their effect on anisotropy. Here, we explore the role of collagen fibers as primary load-bearing components across regions of varying wall thickness, using a three-dimensional representative volume element (RVE) model incorporating explicit collagen fiber architecture and a gradual thickness gradient. We examined two distinct collagen fiber configurations across the thickness transition: one featuring abrupt fiber termination and another with fiber continuity. Finite element analysis (FEA) under uniaxial tension revealed that load transfer by continuous fibers across the specimen markedly reduced the importance of the change in wall thickness, with stretch differentials dropping from ~20% (fiber-termination network) to 0.68% (continuous fibers) and stress differentials dropping from ~65% (fiber-termination network) to 2.3% (continuous fibers). Fiber tortuosity delayed the point at which mechanical response was governed by fiber structure. These findings demonstrate the critical role of fiber continuity in reducing stretch and stress gradients across regions of varying wall thickness and clarify the importance of accurately representing fiber architecture when modeling soft tissues with heterogeneous wall thickness.

## Introduction:

The passive biomechanical properties of arterial tissue are governed by the key constituents of the extracellular matrix (ECM), collagen, elastin, and proteoglycans [1]. Collectively, the ECM components impart the nonlinear and anisotropic mechanical properties of the wall while also serving as a scaffold for the intramural cells. Among these components, collagen fibers serve as the primary load-bearing elements at high stress, playing a central role in regulating tissue stiffness and preventing excessive dilation or rupture by reinforcing the tissue against mechanical deformation.

Advances in imaging techniques such as multiphoton microscopy (MPM) have enabled high-resolution visualization of the three-dimensional architecture of collagen fibers in soft tissues [5]. These imaging techniques have revealed the critical importance of microstructural features such as fiber orientation, density, and tortuosity in determining local mechanical behavior in organs, including arteries, bladder, and cornea [2–8]. However, various pathological conditions such as aneurysms, atherosclerosis and vascular calcifications can disrupt this microstructural organization of the tissue in the form of focal changes wall thickness [9], fiber degradation [10], and abrupt alterations in fiber organization [11, 12]. In particular, regional variations in wall thickness coupled with alterations in fiber organization are frequently observed in both healthy and diseased arterial tissues, yet their impact on the mechanical response remains poorly understood [13–17].

**Figure 1.**
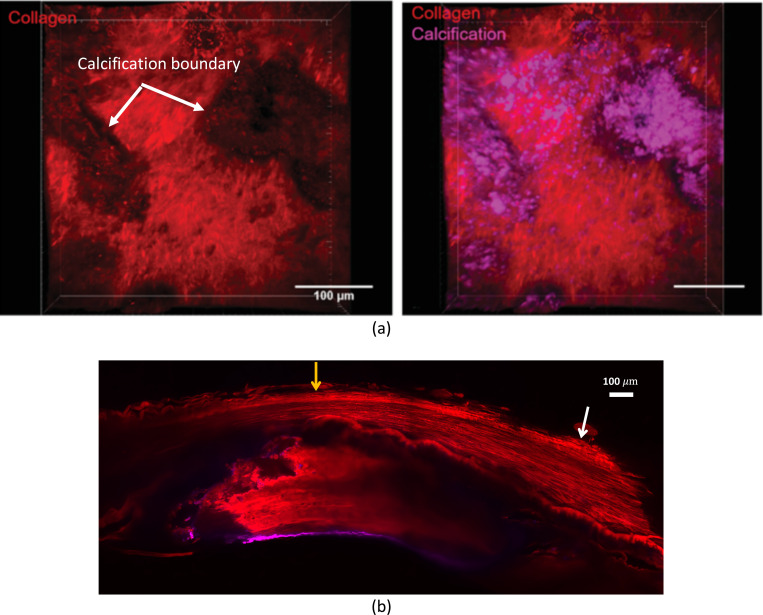
Multiphoton microscopy images showing collagen fiber organization in vascular soft tissues. (a) MPM images from a cerebral aneurysm specimen exhibiting abrupt collagen fiber termination near calcified regions. The left panel shows collagen fibers (red) alone, while the right panel includes both collagen fibers (red) and calcification (magenta), highlighting how mineral deposits disrupt fiber continuity at ECM boundaries (obtained from Gade et al. 2018 [18], with permission). (b) MPM image from cerebral artery tissue with atherosclerotic plaque displaying continuous fibers traversing from a thin region (yellow arrow) to thick region (white arrow), (obtained from Ramezanpour, 2025 [36]). All scale bars are 100 μm.

MPM studies of arterial tissues exhibiting local wall thickness variations have revealed diverse patterns of collagen fiber organization. For example, in cerebral aneurysm tissues, collagen fibers have been observed to terminate abruptly near microcalcification pockets, likely due to structural degradation or disorganization of the ECM (Fig. 1a) [18–20]. In contrast, multiphoton images of the cerebral arterial tissues from our lab (Fig. 1b) show that collagen fibers can also span continuously across thickness gradients. These distinct fiber architectures are expected to yield different mechanical behaviors under loading.

Computational modeling is a valuable tool for investigating how these qualitative differences in fiber organization influence the mechanical response of the tissue. Most prior computational models of arterial biomechanics have either assumed uniform wall thickness [21–24] or utilized isotropic material models - which cannot capture the anisotropic, fiber dominated response inherent to arterial tissues [25–28]. Using these models and the common idealization of homogeneous material properties, thinner regions will experience elevated deformations and stress under mechanical loading, due to reduced cross-sectional areas, a prediction rooted in classical engineering mechanics principles [29, 30]. While recent studies have used continuum anisotropic formulations to capture collagen fiber behavior with local thickness variations, they did not explicitly represent discrete fibers [31–33]. As a result, they did not account for changes in fiber density that naturally arise as continuous fibers cross through regions of varying all thickness.

Fiber tortuosity has been shown to play an important role in determining the biomechanical response of collagenous soft tissues and it is also expected to modulate the contributions of fibers to load bearing in regions of thickness variation [34, 35]. In particular, fibers commonly exhibit undulated morphologies in unloaded collagenous tissues and engage only after surpassing a critical stretch threshold [5]. Although the effects of fiber tortuosity have been studied in tissues of uniform thickness [6], their role in thickness-varying domains has not been analyzed. Thus, it is important to also consider the influence of collagen fiber tortuosity in the present study.

We aim to address this gap in knowledge by explicitly comparing two collagen fiber architectures: one incorporating abrupt collagen fiber terminations (such as identified in the pathological aneurysm tissue in Fig. 1a) and the other with continuous collagen fibers that smoothly transition across regions of varying wall thickness (e.g., Fig. 1b). We hypothesize that collagen fiber spatial continuity across varying thickness regions plays a pivotal role in modulating the mechanical response of soft tissues—challenging the classical notion of the primary role of wall thickness on the stress field. To test this hypothesis, we employed a 3D representative volume element (RVE) model incorporating local thickness variations along with discrete collagen fiber architecture. Finite element analysis (FEA) under uniaxial loading was performed on RVE model to obtain stretch and stress fields. The results demonstrate the importance of identifying the correct collagen architecture when modeling the biomechanical response of soft collagenous tissues. These findings advance our understanding of structure-property function relationships in soft tissues with heterogeneous thickness and provide a better framework for developing more accurate computational models of vascular pathologies.

## 2. Methods

### 2.1 Acquisition and Imaging of arterial microstructure

The contrasting microstructural organizations that are central to the biomechanical comparisons in this study are represented in the two images in Fig. 1. An example with abrupt fiber termination is shown in Fig. 1a and a second with continuous fibers spanning the thickness gradient (arrow) is shown in Fig. 1b.

The specimen in Fig. 1a was harvested from a human cerebral aneurysm, imaged using a multiphoton microscopy (MPM) protocol developed by Gade *et al.* 2019 [18]. Collagen fibers terminate at the boundaries of a region filled with clusters of micro-calcifications. The specimen in Fig. 1b was obtained from a cerebral artery in a study of collagen fiber organization around atherosclerotic plaques using MPM imaging, Ramezanpour 2025 [36]. Here, the fibers can be seen to continuously traverse from thick to thin regions around the plaque (arrow).

### 2.2 RVE Model Construction

**Figure 2:**
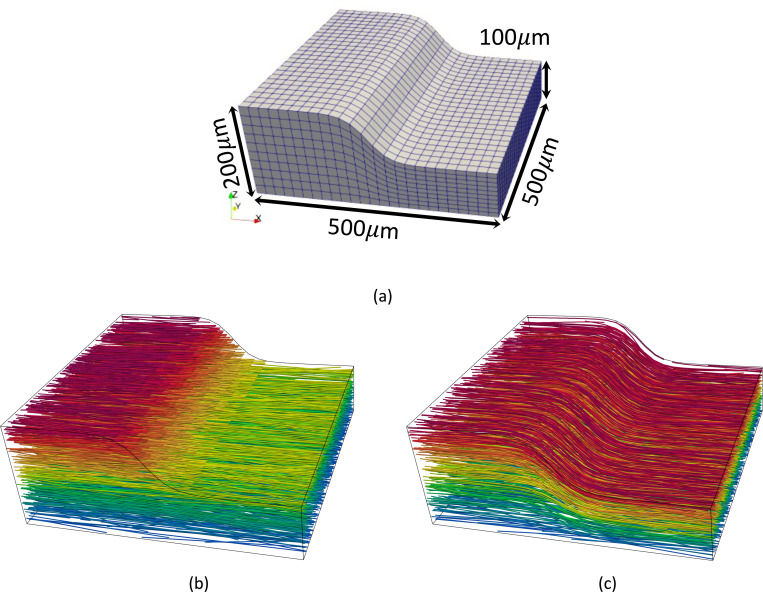
RVE geometry and collagen fiber networks. (a) Hexahedral mesh of the representative volume element (RVE) with a smooth thickness gradient along the x-axis (corresponding to circumferential direction). Thickness transitions from 200 μm (thick region, left) to 100 μm (thin region, right) following a sigmoid function (Eq. 1). The mesh comprises 500×500 μm in-plane dimensions with hexahedral elements. (b) Collagen fiber network shown with abrupt termination at the thickness transition boundary. (c) Smoothly transitioning fiber network shown where continuous collagen fibers span the thickness gradient. Fibers adapt spatially to maintain smooth continuity in regions of thickness gradient.

A three-dimensional representative volume element was developed to investigate the biomechanical effect of the wall thickness variation. The specimen geometry was designed as a rectangular base of dimensions of 500 × 500 μm with thickness varying smoothly from 100 μm (thin region) to 200 μm (thick region) along the uniaxial tensile loading direction (x-axis as shown in Fig. 2a). The thickness profile was mathematically defined using a sigmoid function to ensure gradual transition as shown in Eq. 1.

(1) $$t(x)=t_{\min}+\frac{t_{\max}-t_{\min}}{1+e^{a(x+b)}}$$

where t_min_ = 100 μm, t_max_ = 200 μm, and parameters a (scaling) and b (shifting) controlled the steepness and position of the thickness gradient. A planar quadrilateral mesh was first generated in Cubit (Coreform LLC, Orem, UT) and extruded along the thickness direction (z-axis) using a custom Python script to assign spatially varying element thickness based on the sigmoid function. The resulting hexahedral mesh (Fig. 2a) ensured element continuity across the thickness transition region.

### 2.3 Collagen Fiber Network Generation

Discrete fibers were modeled using a fiber-embedded finite element technique developed by our group [11, 35, 37]. Collagen fibers were modeled as 1D rod elements where fibers were primarily aligned along the uniaxial loading direction (x-axis as shown in Fig. 2a). To mimic the physiological fiber dispersion in the arterial tissue, the individual fiber orientations were randomly generated from a Gaussian distribution with standard deviation of around 10° [38, 39]. The collagen fiber diameters were set to 1.28 μm based on multiphoton microscopy measurements [40]. To understand the effect of collagen fiber continuity in thickness transition regions on the mechanical behavior, two distinct collagen fiber networks were implemented in our RVE model and were subjected to uniaxial tensile loading. In particular, these network models are:

1. **Abrupt Fiber Termination Network**: In this configuration, collagen fibers were restricted to x-y planes (see Fig. 2a) throughout the RVE thickness and did not continue across the region of thickness variation. A random ray-shooting algorithm was used to generate fibers in which the random seed points were selected within the RVE domain, and rays were extended from those seed points within the x-y plane with fiber dispersion until they intersected the RVE boundary. This process was repeated until the desired fiber volume density of 35.5% was reached [6]. As a result, fibers originating in the thick region terminated abruptly at the edge of the thickness gradient, producing a discontinuous fiber architecture representative of structurally disrupted tissue (Fig. 2b).
2. **Continuous Fiber Transition Network**: In contrast, this network incorporated fibers that smoothly traversed from the thick to thin region across the thickness gradient, representing fiber continuity across the specimen. During RVE mesh extrusion along the z-axis, 30 intermediate non-planar surfaces were extracted to capture the smoothly varying thickness profile. Fibers were then generated as connected line segments across these layers, aligned primarily along the x-axis (see Fig. 2a) with angular dispersion to maintain orientation distribution. This method ensured that individual fibers gradually transitioned from thick to thin regions (and vice versa), preserving fiber continuity across the thickness gradient (Fig. 2c). The total number of fibers was calibrated to achieve a collagen volume density of 35.5% in the RVE insuring consistent fiber density with the abrupt fiber termination network.

### 2.4 Finite Element Analysis

Fibrous components of the tissue were modeled as 1D rod elements representing collagen fiber segments embedded within 3D 8-noded hexahedral elements representing non-fibrous matrix. Affine deformation between discrete fibers and the non-fibrous component was ensured in the model throughout the domain with no-slip condition at the fiber-continuum interface. The constitutive model of the non-fibrous component was defined as the nearly incompressible two-parameter neo-Hookean material model shown in Eq. 2.


(2) $$\Psi =0.5\mu \left(J^{-\frac{2}{3}}I_{1}-3\right)+0.5K{\left(J-1\right)}^{2}$$

Here, Ψ denotes the strain energy per unit reference volume, μ represents the shear modulus, J denotes the determinant of the deformation gradient, K is a penalty parameter that enforces the incompressibility condition, and I_1_ refers to the first invariant of the right Cauchy-Green deformation tensor. The rods were modeled as 1D NeoHookean elements with a recruitment stretch built in, as described in [6, 35]. Briefly, the constitutive model for the fibrous component was defined such that fiber stress $\left(P_{f}\right)$ increases linearly with fiber stretch beyond the recruitment stretch $\lambda_{r}$ with elastic modulus of $E_{f}$ as shown in Eq. 3 [6]. In this study, the mechanical response was compared for two recruitment stretches (${\text{λ}}_{r}$ =1.01, 1.25) in order to understand the combined effect of fiber tortuosity and fiber organization in thickness varying tissues.


(3) $$P_{f}=\left\{\begin{array}{cc} 0 & \text{if}\lambda_{f}<\lambda_{r}\Rightarrow \lambda_{t}<1\\ E_{f}\left(\lambda_{f}/\lambda_{r}-1\right) & \text{if}\lambda_{f}\geq \lambda_{r}\Rightarrow \lambda_{t}\geq 1 \end{array}\right.$$

To simulate uniaxial tensile testing, displacement-controlled boundary and loading conditions were applied on our computational model as per our previous publication [6]. Simulations were performed quasi-statically with 2000 load steps to ensure convergence.

### 2.5 Data Analysis

All displacement results were post-processed in Paraview (Kitware Inc., Clifton Park, NY) to obtain the localized stretch in the uniaxial loading direction and von Mises’s stress fields in all thickness regions. Regional average properties were computed for thick (t = 200 μm) and thin (t = 100 μm) regions of the RVE. Percent differences in average stretches in the loading direction between regions were calculated as:

(4) $$\text{Δ}\lambda (\text{\%})=\frac{\lambda_{thin}-\lambda_{thick}}{\lambda_{thick}}\times 100$$

## Results:

### 3.1 Stretch and Stress Patterns Under Uniaxial Loading

**Figure 3:**
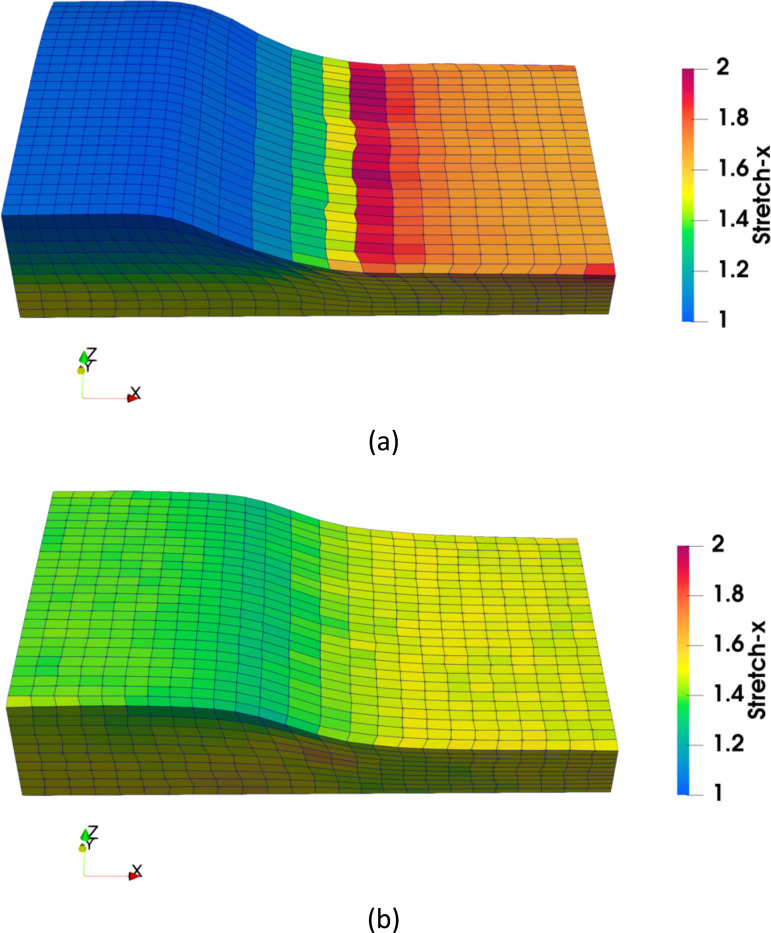
Spatial distribution of stretch in the loading direction at an applied tissue stretch λ = 1.5 for (a) Abrupt fiber network with average stretch of 1.3 ± 0.18 in thick and 1.56 ± 0.06 in thin region. (b) Continuous fiber network with average stretch of 1.47 ± 0.07 in thick and 1.5 ± 0.04 in thin region.

**Figure 4:**
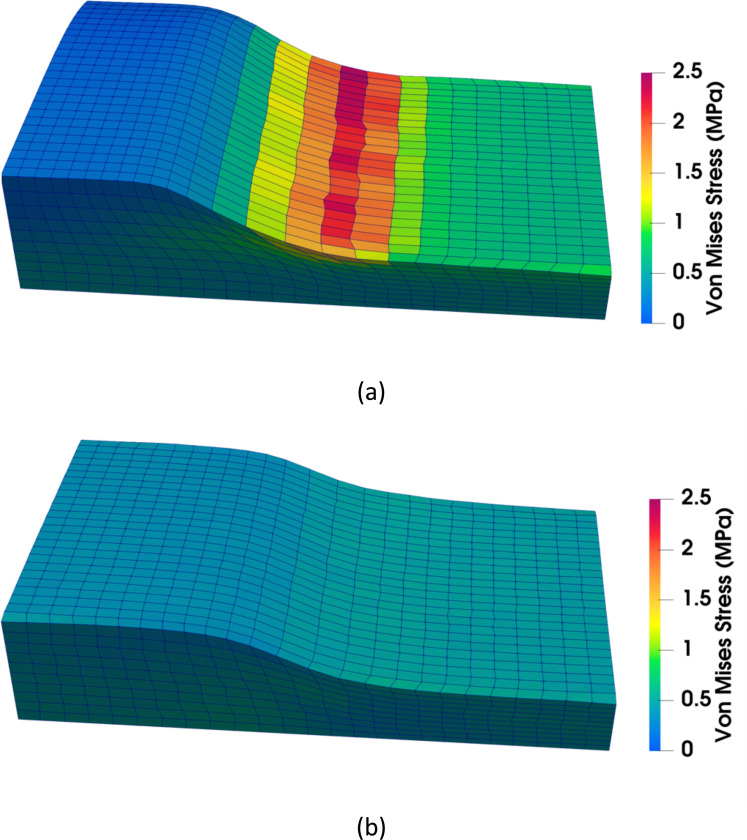
Spatial distribution of von Mises stress at applied tissue stretch λ = 1.5 for (a) Abrupt fiber network with average stress of 0.35 ± 0.15 MPa in thick and 0.57 ± 0.18 MPa in thin regions. (b) Continuous fiber network with average stress of 0.44 ± 0.05 MPa in thick and 0.45 ± 0.16 MPa in thin regions.

Finite element simulations of the RVE under uniaxial tensile loading revealed distinct stretch and stress patterns between the abrupt and smooth fiber networks. At an applied tissue stretch of λ=1.5, the abrupt fiber termination network exhibited substantial stretch and stress disparities between thick (t=200 μm) and thin (t=100 μm) regions (Figs. 3a & 4a). The thin region experienced an average stretch of 1.56 ± 0.06, accompanied by a relatively high von Mises stress of 0.56 ± 0.10 MPa. In contrast, the thick region experiences a lower stretch (1.3 ± 0.18) and stress (0.34 ± 0.15 MPa). The maximum von Mises stress across the entire RVE tissue with abrupt fiber network was 2.49 MPa.

In contrast, the continuously smooth fiber transition network diminished the spatial gradient in the mechanical response (Figs. 3b & 4b). The thin region exhibited an average stretch of 1.48 ± 0.02 and average stress of 0.45 ± 0.02 MPa, while the thick region showed an average stretch of 1.47 ± 0.07 and average stress of 0.44 ± 0.05 MPa. The maximum von Mises stress in the RVE tissue with continuous fiber network was 0.664 MPa. The percent difference in stretch between the two constant thickness regions decreased from 20% for the abrupt fiber termination network to 0.68% for the continuous fiber network.

**Figure 5:**
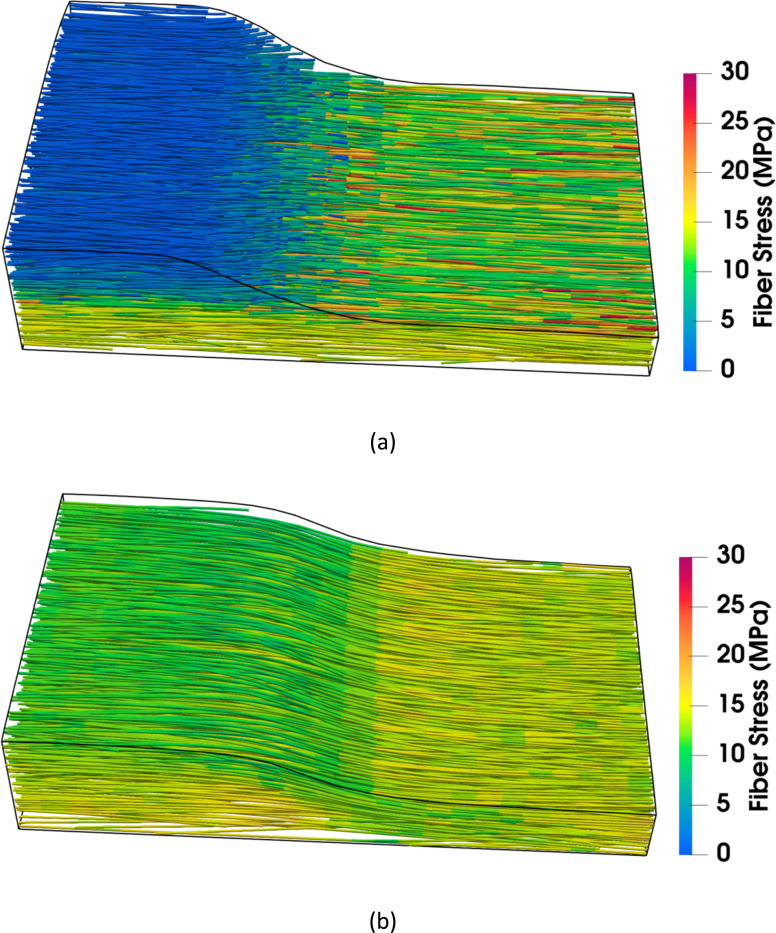
Spatial distribution of stress within individual fibers under uniaxial loading at the applied tissue stretch λ = 1.5 for (a) Abrupt fiber termination network and (b) Continuous fiber transition network. In the abrupt network (a), the maximum fiber stress reaches 30 MPa, with an uneven stress distribution characterized by lower stress in the thick region. In contrast, the continuous network (b) exhibits a lower maximum fiber stress of 20 MPa and a more uniform stress distribution across the RVE. Fibers in this network transition smoothly between thick and thin regions, facilitating consistent load transfer. These results highlight the mechanical advantage of fiber continuity in reducing stress concentrations and promoting uniform mechanical response in tissues with wall thickness gradients.

Fiber-level stresses at λ = 1.5 are shown in Fig. 5. In the abrupt network, collagen fibers in the thin region experienced substantially higher stress (14.32 ± 2.08 MPa) compared to those in the thick region (7.67 ± 6.15 MPa) (Fig. 5a). Notably, the continuous network exhibited a more uniform fiber stress distribution, with the thin and thick regions experiencing similar values of 9.83 ± 6.23 MPa and 9.55 ± 6.09 MPa, respectively (Fig. 5b).

### 3.2 Local Collagen Fiber Volume Density

**Figure 6:**
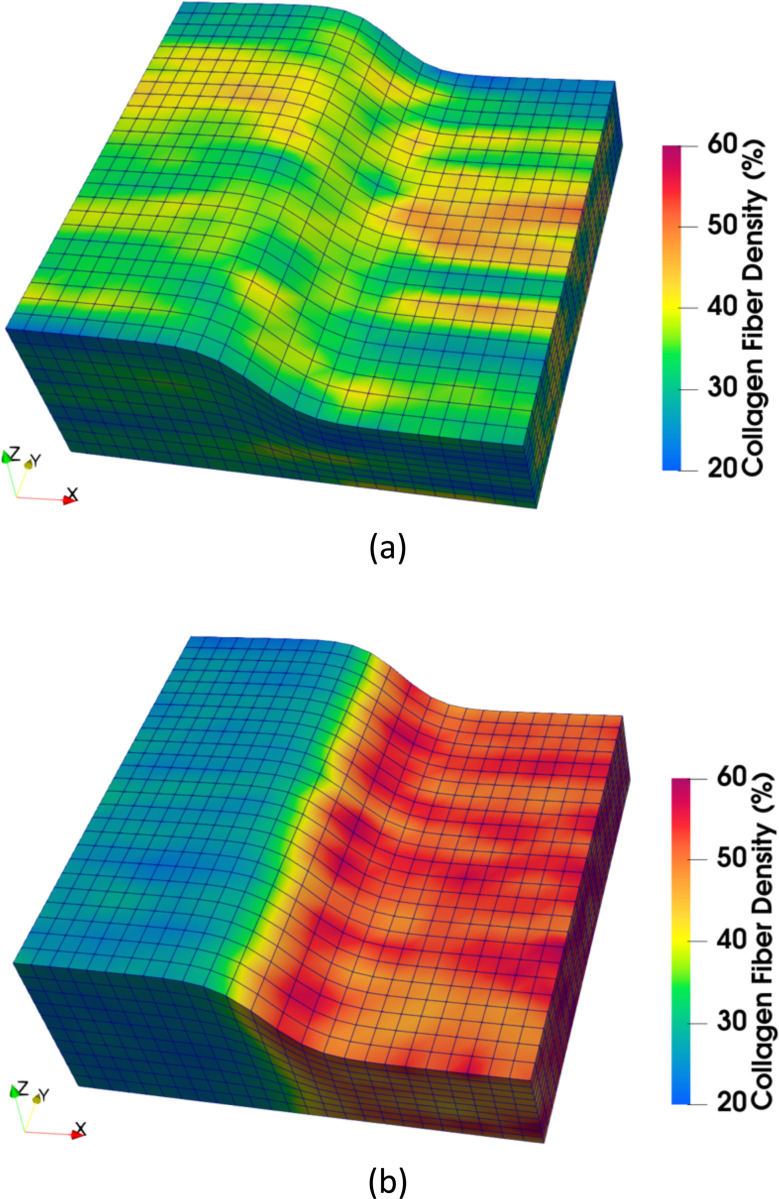
Distribution of collagen fiber volume density in the RVE for (a) Abrupt fiber termination network and (b) Continuous fiber transition network. In the abrupt network (a), a fiber volume fraction of 35.5% was prescribed. In contrast, as the continuous network (b) preserves the same total number of fibers across all thickness regions, the thinner region has higher fiber density due to its smaller wall thickness.

The fiber volume density will naturally vary across the thick and thin regions in specimens for which fibers are continuous, Fig. 1b. In particular, while the overall collagen volume density was maintained at 35.5% for both fiber architectures, local density variations emerged due to differences in fiber arrangement. To quantify this, a local fiber density analysis was performed, Fig. 6. In the abrupt fiber termination model, the fiber volume fraction was 35.31 ± 5.02% in the thin region and 35.20 ± 3.61% in the thick region (Fig. 6a). In contrast, in the continuous fiber network model, the same number of fibers traversed the RVE, resulting in a higher fiber volume fraction in the thin region (51.93 ± 2.19%) compared with the thick region (26.04 ± 1.10%), Fig. 6b.

### 3.3 Influence of Fiber Tortuosity on Differences in Deformation and Stress Fields

**Figure 7.**
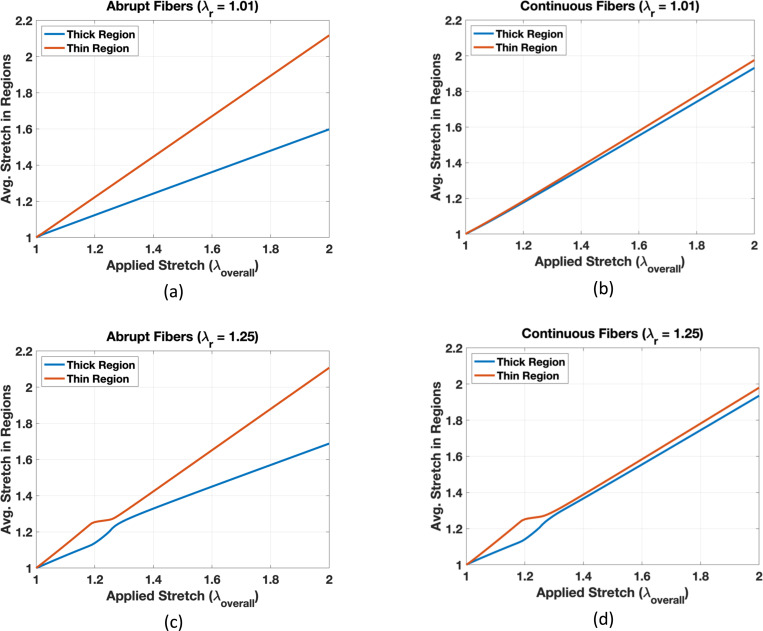
Deformation disparities between thick and thin regions as a function of applied stretch. Average stretch in thick (blue lines) and thin (orange lines) regions for abrupt and smooth fiber networks at tortuosity thresholds λ_r_ = 1.01 (almost no tortuosity) (a-b) and λ_r_ = 1.25 (c-d).

Fiber tortuosity (controlled through specification of recruitment stretch, λ_r_) substantially modulated the low stretch deformation behavior (Fig. 7). For λ_r_ = 1.01, fibers start engaging almost immediately after applying the external load. Consequently, for the abrupt fiber termination network, the stretch difference in thick and thin regions keeps widening, reaching 32.5% at applied tissue stretch of two. For the continuous fiber network, there is a marginal stretch difference of only 2.27% at the applied tissue stretch of two.

For λ_r_ = 1.25, fibers engaged later due to higher undulation associated with the larger recruitment stretch. Prior to fiber recruitment in both thick and thin regions, (λ < 1.25), the abrupt fiber termination network showed a 10.56% higher stretch in the thin region compared to the thick region. In contrast, once fibers in both regions were activated (λ ≥ 1.25), the stretch difference reduced to 3.75% as primary load transfer shifted from the underlying matrix to the stiffer collagen fibers. Similarly, in the continuous fiber network, the disparity decreased from 10.06% (pre-recruitment) to 1.49% post-recruitment.

Notably, for the abrupt fiber termination network, post-recruitment differences in average stretch between thick and thin regions began to re-emerge at higher values of applied tissue stretch. For example, the stretch disparity increased to 24.82% at λ = 2. In contrast, the continuous fiber network maintained a smaller difference (2.3%) between thick and thin regions.

## 4. Discussion

This study aimed to discern the importance of fiber continuity in regions of thickness gradient and how this influence is modulated by fiber tortuosity—under uniaxial loading. The results challenge the conventional view that stress heterogeneity in arterial tissue is largely determined by geometric variations, instead highlighting the pivotal role of collagen microstructure in determining the stress and strain fields.

### 4.1 Collagen Fiber Continuity Mitigates Geometry-Induced Stretch and Stress Disparities

The finite element simulations demonstrated that when collagen fibers smoothly transition through regions with a thickness gradient, they substantially attenuate the stretch disparity between thin and thick regions of the arterial wall. In networks with abrupt fiber terminations, thin regions experienced markedly elevated stretches, reaching up to 20% higher strain compared to thicker counterparts at the overall tissue stretch of two. This result confirms intuitive mechanical expectations wherein thinner regions have greater stress and strain —due to diminished area and larger stress. However, when collagen fibers were allowed to transition smoothly across thickness gradients, the disparity dropped dramatically to 0.68% under the same loading conditions. This more homogeneous strain distribution can be attributed to the ability of the continuous fibers to transfer load from thick to thin regions, thereby redistributing mechanical stresses across the tissue. While the examples shown in Fig.1 are for vascular tissues, these findings are applicable more generally to collagenous soft tissues.

Notably, these results demonstrated that tissues with fiber continuity behave similarly to constant-thickness tissues, after fibers are recruited. These findings suggest that, when fiber continuity is confirmed but local thickness measurements are unavailable, constant-thickness models may still provide reasonably accurate mechanical predictions. Such an observation has important implications for finite element analysis of diseased vessels, as many in-vivo imaging modalities—such as digital subtraction angiography (DSA) or MRI—lack the resolution needed to capture precise local thickness variations. In these cases, accurate failure prediction through finite element analysis may still be possible without explicitly modeling thickness variation, provided that fiber continuity is present in the tissue.

Interestingly, Cavinato *et al.* (2019) have shown that there is no significant correlation between thickness variations and ascending thoracic aneurysm rupture [41]. Such findings could be explained if collagen fibers smoothly transitioned across the thickness gradients, suggesting a physiological role for fiber continuity in mechanical resilience. Notably, modeling efforts or clinical interpretations based on geometric parameters alone may be inappropriate for estimating local mechanical vulnerabilities, especially in pathological settings like aneurysms or dissections.

### 4.2 Fiber Volume Density Distribution and Implications for Load Sharing

Variations in fiber density arise naturally when fibers smoothly transition between regions of different thickness, as illustrated in Fig. 6. Therefore, fiber density is directly coupled to fiber organization (terminated versus continuous) and is an important mechanism by which fiber organization shapes the tissue deformation and stress fields. In the abrupt fiber network (Fig. 6a), fibers terminate at the transition between thick and thin regions, resulting in more fibers occupying the thicker region and fewer in the thinner one. This leads to approximately uniform fiber density across both regions. The reduced number of fibers in the thin region diminishes its load bearing contributions, amplifying local stress and stretch.

In contrast, in the continuous fiber network, the same number of fibers span all thickness regions due to fiber continuity. As wall thickness decreases, the confined volume in the thin region causes a local increase in fiber density (Fig. 6b). This densification enhances the mechanical support of the thin region, facilitating improved load sharing and contributing to a more uniform deformation response across the tissue. This effect emerges directly from fiber continuity, enabling a more balanced mechanical response in thickness-varying arterial walls.

### 4.3 Fiber Tortuosity Introduces Transitional Mechanical Behavior

The simulations further revealed that fiber tortuosity also substantially modulates the local stretch distribution. In the pre-recruitment phase (λ < λ_r_), the mechanical response was predominantly governed by the geometry of the RVE as the isotropic matrix was the sole load bearing component. As a result, there were substantial stretch disparities in regions of differing wall thickness for both fiber architectures. For instance, in the continuous fiber network with λ_r_ = 1.25, the thin region stretched 10.06% more than the thick region prior to fiber engagement. Once the fiber stretch exceeded the recruitment threshold in both regions, collagen fibers began to engage and assume the primary load-bearing role, substantially reducing the stretch disparity to below 5%. This highlights the dual influence of fiber tortuosity: initially delaying fiber recruitment and amplifying geometry-driven deformation, and later facilitating redistribution of stretch and stress as fibers straighten and stiffen.

Following fiber recruitment, the mechanical response becomes predominantly governed by fibers. However, even in the continuous fiber network, minor stretch disparities in both regions persist at higher stretches, though these are substantially smaller compared to the abrupt fiber termination network. This difference indicates that, while continuous fiber network enhances mechanical uniformity, geometric effects are not entirely eliminated. This can be attributed to loading within the isotropic matrix in which thicker regions have lower stress due to the larger cross-sectional area.

Importantly, since failure in arterial tissues is generally initiated by fiber rupture—which occurs only after fiber recruitment— a constant thickness idealization may be reasonable in studies of tissue failure when fiber continuity can be confirmed. Namely, once the fibers are engaged, the geometry-driven disparities diminish, and the dominant fiber-governed mechanics can be captured reasonably well without explicitly modeling local thickness variation.

### 4.4 Limitations and Future Directions

While this study provides valuable mechanistic insight, there are some limitations in the study that drive future directions of investigation. First, an idealized geometry was chosen with smooth, mathematically defined thickness gradients and simplified boundary conditions. This idealization was chosen to allow a controlled investigation of microstructural effects. In future studies, we will build on the present work to investigate the additional effects of geometric complexity and heterogeneity found in tissues such as cerebral aneurysms and atherosclerotic arteries. Future studies will also explore the role of residual stress states and multiaxial in vivo loading conditions.

This study focused on two idealized extremes of fiber architecture in thickness-varying tissue: one in which all collagen fibers span continuously across the thickness transition region, and another in which fibers terminate abruptly at the boundaries of that region. In native tissues, a hybrid presentation may exist where a portion of fibers transition smoothly, while others terminate abruptly within the same region. Future studies could incorporate such mixed fiber architectures to investigate how varying the ratio of continuous to abruptly terminating fibers influences the mechanical response.

Moreover, although the collagen fiber network was generated using physiological parameters, experimental validation of the simulated mechanical response—including regional stretch and stress distributions is an important area for future investigations. The predictive power of the framework could be examined using mechanical testing with local strain measurement techniques such as digital image correlation (DIC), combined with multiphoton imaging.

## 5. Conclusion

This study highlights the critical role of continuity of fibers in modulating the mechanical behavior of collagenous soft tissues with wall thickness variations. Finite element simulations revealed that stretch and stress disparities between thick and thin regions can be ameliorated in specimens with continuous fibers spanning regions of thickness gradients, through effective load transfer and localized increases in fiber density. Fiber tortuosity modulates this behavior by delaying the influence of collagen fibers to higher stretches. As a result, before fiber recruitment, the load is borne by the underlying matrix and hence dominated by the effect of local wall thickness. These findings demonstrate that geometry alone cannot explain local mechanics. Rather, microstructural fiber organization plays a dominant role once fibers are recruited. Therefore, accurate representation of collagen structure is vital for reliable biomechanical modeling and rupture risk prediction, underscoring the need for imaging tools that capture microstructural features such as fiber continuity and local density. Notably, for tissues with continuous fibers, constant-thickness models may yield accurate predictions, even when local thickness data are unavailable.
